# Identification of a Continuously Stable and Commercially Available Cell Line for the Identification of Infectious African Swine Fever Virus in Clinical Samples

**DOI:** 10.3390/v12080820

**Published:** 2020-07-28

**Authors:** Ayushi Rai, Sarah Pruitt, Elizabeth Ramirez-Medina, Elizabeth A. Vuono, Ediane Silva, Lauro Velazquez-Salinas, Consuelo Carrillo, Manuel V. Borca, Douglas P. Gladue

**Affiliations:** 1 ARS, USDA, Plum Island Animal Disease Center, Greenport, New York, NY 11944, USA; Ayushi.Rai@usda.gov (A.R.); Sarah.Pruitt@usda.gov (S.P.); Elizabeth.Ramirez@usda.gov (E.R.-M.); Elizabeth.Vuono@usda.gov (E.A.V.); Ediane.Silva@usda.gov (E.S.); Lauro.Velazquez@usda.gov (L.V.-S.); 2 Oak Ridge Institute for Science and Education (ORISE), Oak Ridge, TN 37830, USA; 3 Department of Pathobiology and Population Medicine, Mississippi State University, P.O. Box 6100, Starkville, MS 39762, USA; 4 Department of Anatomy and Physiology, Kansas State University, Manhattan, KS 66506, USA; 5 APHIS, USDA, Plum Island Animal Disease Center, Greenport, New York, NY 11944, USA; Consuelo.Carrillo@usda.gov

**Keywords:** ASFV, ASF, diagnostics

## Abstract

African swine fever virus (ASFV) is causing outbreaks both in domestic pigs and wild boar in Europe and Asia. In 2018, the largest pig producing country, China, reported its first outbreak of African swine fever (ASF). Since then, the disease has quickly spread to all provinces in China and to other countries in southeast Asia, and most recently to India. Outbreaks of the disease occur in Europe as far west as Poland, and one isolated outbreak has been reported in Belgium. The current outbreak strain is highly contagious and can cause a high degree of lethality in domestic pigs, leading to widespread and costly losses to the industry. Currently, detection of infectious ASFV in field clinical samples requires accessibility to primary swine macrophage cultures, which are infrequently available in most regional veterinary diagnostic laboratories. Here, we report the identification of a commercially available cell line, MA-104, as a suitable substrate for virus isolation of African swine fever virus.

## 1. Introduction

African swine fever virus (ASFV) is the only member of the virus family Asfarviridae, and is the etiological agent that causes African swine fever (ASF). This large double-stranded DNA virus has more than 150 open reading frames (ORFs) that are encoded in the 180–190 kilobase genome. ASF disease can range from sub-clinical to lethal depending both on the specific host that is infected and the specific strain of virus [1]. Several sub-Saharan African countries and Sardinia (Italy) have endemic ASF. Recent outbreaks of ASF started after a single introduction of ASFV in the Caucasus region in 2007. This outbreak affected Georgia, Armenia, Azerbaijan, and Russia, and more recently has spread as far west as Poland, heightening the concern that this disease could spread into neighboring European countries. Importantly, the disease has spread east, reaching China, and has continued to spread to other southeast Asian countries including Mongolia, Vietnam, Cameroon, North and South Korea, Laos, Myanmar, Philippines, Timor-Leste, Indonesia, Papua New Guinea, and India. The current outbreak strain is highly contagious and, in domestic pigs, it is highly lethal, causing up to a 100% mortality rate, particularly in experimental conditions. Due to the potential widespread loss of domestic pigs, the US swine industry could suffer from substantial economic losses should an outbreak occur [2].

There are no vaccines currently available to prevent ASF [1], and control of outbreaks has relied on the quarantine and culling of infected or exposed animals. Diagnosis of ASFV in clinical samples, primarily whole blood, is done using real-time polymerase chain reaction (rt-PCR), a molecular test that detects small parts of the viral genome but cannot detect live infectious virus. Virus isolation is necessary for the confirmation of active infection and downstream analyses, such as whole genome sequencing; currently, virus isolation can only be performed using primary swine macrophages. Production of primary swine macrophages is very time and labor consuming since cells need to be collected from swine blood or isolated from the lungs [3]; these cultures are often not readily available in most veterinary diagnostic laboratories.

Previous studies by us and others have shown that ASFV only replicates in established cell lines once the virus adapts in those particular cell lines, usually after a process involving successive passages [4,5,6]. To date, no established commercially available cell line has been shown to be suitable for ASF virus isolation using field samples. In this study, we identified a cell line that is capable of supporting the detection of ASFV in field samples with a TCID_50_ sensitivity comparable to that of primary swine macrophages. A robust screening effort of commercially available cell lines resulted in the identification of African green monkey cells MA-104 (ATCC# CRL-2378.1) as a substitute for primary swine macrophages for ASFV virus isolation.

## 2. Materials and Methods

### 2.1. Ethics Statement

Animal experiments to collect blood for swine macrophages were performed under biosafety level 3AG conditions in the animal facilities at Plum Island Animal Disease Center (PIADC), Orient Point, NY. All experimental procedures were carried out in compliance with the Animal Welfare Act (AWA), the 2011 Guide for Care and Use of Laboratory Animals, the 2002 PHS Policy for the Humane Care and Use of Laboratory Animals, and the U.S. Government Principles for Utilization and Care of Vertebrate Animals Used in Testing, Research, and Training (IRAC 1985), as well as specific animal protocols reviewed and approved by the PIADC Institutional Animal Care and Use Committee of the US Departments of Agriculture and Homeland Security (protocol number 205.03-17-R, 09-28-17).

### 2.2. Cell Culture and Viruses

Primary swine macrophage cell cultures were prepared from defibrinated swine blood as previously described [7], were then reseeded into Primaria 6- or 96-well tissue culture plates at a density of 5 × 10^6^ cells per ml using macrophage media as previously described [7], and were incubated at 5% CO_2_ at 37 °C to be used 24 h later. MA-104 cells were obtained by American Tissue Culture Collection (ATCC) (catalog number ATCC CRL-2378.1). Historically, MA-104 cells contained a mix culture from two different species of monkeys. The clone used in this study was derived from single cells and was determined to be a cell derived from Cercopithecus aethiops. The full name of the cell line available on ATCC is MA-104 Clone 1, which is characterized as having an epithelial cell origin. MA-104 cells were plated in 6- or 96-well plates with new media at a density of 5 × 10^6^ and used in assays immediately using Eagle’s Minimum Essential Medium, 1X antibiotic-Antimycotic (Fisher Scientific Waltham, MA, USA; Catalog #15-240-096), and 10% fetal bovine serum, and then were incubated at 5% CO_2_ at 37 °C. Ma104 cells were subculture when confluent at ratios 1:3 to 1:10; confluent flasks were used by detaching the cells using 0.25% Trypsin 0.53 mM EDIA solution, and were reseeded as recommended by ATCC. ASFV Georgia (ASFV-G) was a field isolate kindly provided by Dr. Nino Vepkhvadze from the Laboratory of the Ministry of Agriculture (LMA) in Tbilisi, Republic of Georgia. The ASFV E70 virus was provided by J.M Escribano from INIA, Madrid (Spain). The other ASFV isolates tested in this study are part of the Plum Island Animal Disease Center Reference collection: Warthog (NCBI Accession number: AY261366.1) collected before 2003 from an infected warthog, Ba71 (NCBI Accession number KP055815) isolated in Spain (1971–1975) [8], Pretoriuskop/96/4 (Pret4) (NCBI Assession number #AY261363) collected in 1996 in South Africa, Kruger National Park, Kerita (KE); Victoria Falls (VI), Uganda95 (UG), Zimbabwe95 (ZM), and Tengani (TE) [9] are part of the reference collection but have not been fully sequenced. At one hour post-infection (hpi), 100 µL of 25% *v*/*v* suspension of red blood cells were added to each well. Cells were observed daily for the presence of Hemoadsorption (HA).

### 2.3. Hemoadsorption in Swine Macrophages

Hemadsorption (HA) is the ability of ASFV-infected cells to form rosettes in the presence of swine red blood cells, and it is an easy visual test for detecting infectious ASFV in cell culture. MA-104 cells were plated at a density of 1 × 10^6^ cells/mL in 6-well tissue culture dishes. Cells were infected using a MOI of 1 with the current ASFV epizootic strain Georgia/2007 (a genotype II stain) or ASFV strain Ba71 (a genotype I strain). At one-hour post-infection (hpi), 100 µL of 25% *v*/*v* suspension of red blood cells were added to each well.

### 2.4. Virus Titration

Swine macrophages were plated the day before at 1 × 10^6^ cells per 96-well plate, while MA-104 cells were plated at a similar concentration and were used immediately after plating. Ten-fold serial dilutions were prepared from each archived sample using macrophage media for each cell type. The cells were then incubated at 37 °C with 5% CO_2_, and 24 h later, 1 µL of 30% red blood cells was added to each well. After seven days of incubation, the presence of the infected cells was detected by HA, and titers were calculated using the Reed and Muench method by Hemeadsorbing dose (HAD) and were expressed as log_10_ HAD_50_/mL.

### 2.5. Extraction of ASFV DNA and RtPCR of ASFV

ASFV viral DNA was extracted using the MagMAX™ Pathogen RNA/DNA Kit (ThermoFisher Scientific, Waltham, MA) according to the manufacturer′s instructions. Rt-PCR was performed as described by Zsak et al. [10], with small modifications. Briefly, the PCR master mix was composed of 5 μL of template DNA, 0.25 μM of each primer, 0.25 μM of each probe, and 10 μL of 2X iQ™ Multiplex Powermix (Bio-Rad, Hercules, CA, USA). The parameters for thermocycling starts with a denaturation step at 95 °C for 10 min, followed by 45 cycles of denaturation at 95 °C for 15 s, and annealing/extension at 65 °C for 45 s using an Applied Biosystems ABI 7500 Fast Real-Time PCR System (ThermoFisher Scientific).

### 2.6. Immunoproxidase Staining for ASFV

MA-104 cells were plated at a density of 1 × 10^6^ cells/mL in 6-well tissue culture dishes, and after 24 h in the culture, cells were fixed with acetone and methanol (50:50) for 15 min. Viral infectivity was assessed using an immunoproxidase assay with an ASFV p30 monoclonal antibody [4] at a 1:200 dilution using a Vectastain ABC kit (Vector Laboratories, Burlingame, CA, USA).

## 3. Results

### 3.1. ASFV-Infected MA-104 Cells Can Be Detected by Hemadsorption (HA)

**Figure 1:**
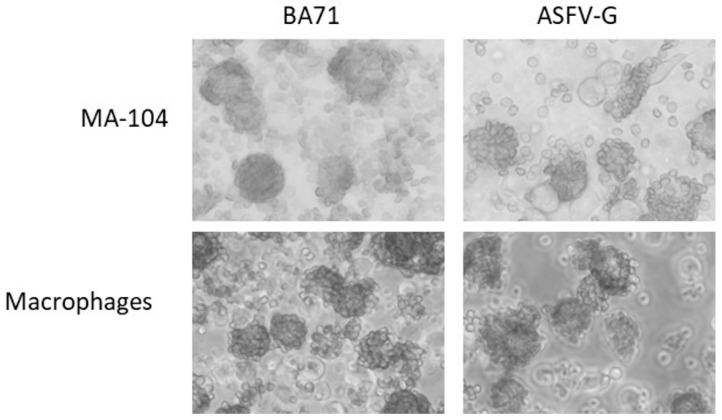
MA-104 cells were infected with the indicated virus in the presence of red blood cells. Hemadsorption was observed 24 h after infection.

Hemadsorption (HA) is the ability of ASFV-infected cells to form rosettes in the presence of swine red blood cells, and it is an easy visual test for detecting infectious ASFV in cell cultures. Twenty-four after infected with either ASFV-G or BA71, both MA-104 cells and primary swine macrophages cells were observed for hemadsorption (HA) as an indicator of the presence of ASFV-infected cells (Figure 1). Results demonstrate that ASFV-infected MA-104 cells form rosettes with a very similar phenotype to those rosettes that are typically observed in infected macrophages [11,12,13].

### 3.2. ASFV-Infected MA-104 Cells Can Be Specifically Stained by Immunocytochemistry Using an Anti-ASFV p30 Monoclonal Antibody

**Figure 2:**
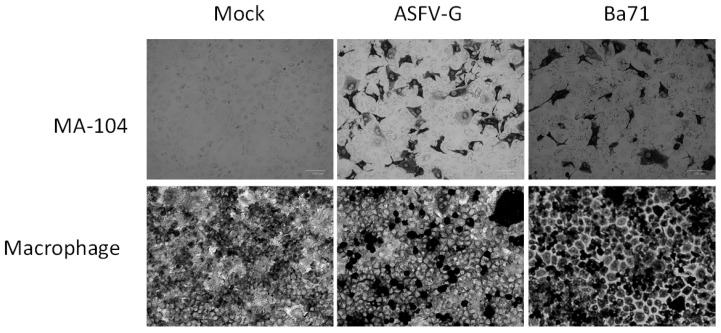
MA-104 cells infected or mock infected with the indicated virus. The presence of the virus was determined by using a monoclonal antibody that detects ASFV protein p30, and it was visualized using an immunoproxidase assay.

HA relies on the presence of a functional CD2-like gene in the ASFV genome. Some natural isolates have been shown to have mutations in CD2 that alters the expression of a functional protein, and they are unable to form rosettes [12]. Therefore, we assessed if MA-104 cells could be used as a substrate for the detection of infectious ASFV using immunocytochemistry with a monoclonal antibody specifically recognizing ASFV protein p30as described in the Material and Methods. MA-104 showed very clear positive staining (Figure 2) when infected with either virus strain, demonstrating that MA-104 could be used as an alternative substrate for staining of non-HA ASFV strains. When tested using the same immunocytochemistry technique, swine macrophages had a consistent high background. This made results difficult to interpret.

### 3.3. Relative Sensitivity of MA-104 Cells to Detect Infectious ASFV from Different Field Isolates Compared to that of Primary Swine Macrophages or Real-Time PCR

**Figure 3:**
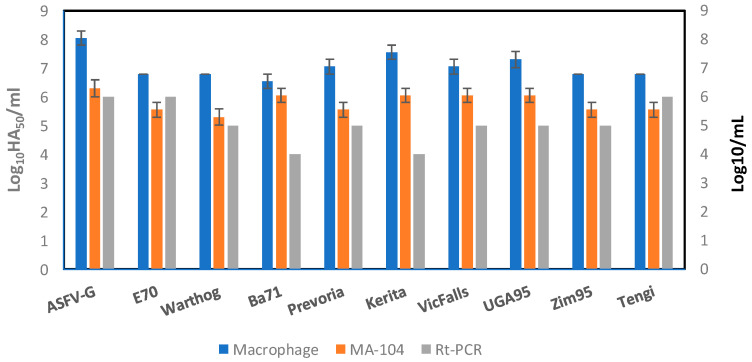
Comparison of the titration of African Swine Fever Virus (ASFV) isolates in swine macrophages (blue) or MA-104 (orange) cells with real-time PCR. Titrations are expressed in log_10_ HA_50_/mL, the error bars are the standard deviation between replicates, and rt-PCR is expressed as L]log_10_/mL, indicating the last dilution that had a positive CT value for ASFV.

The relative sensitivity of MA-104 cells to detect infectious ASFV was evaluated in comparison to that of primary swine macrophages. The amount of infectious virus detected in archived clinical samples (from our ASFV collection) containing different ASFV field isolates were quantified by virus titration performed in parallel in both swine primary macrophage cells and MA-104 cells. Swine macrophages and MA-104 cells were plated at a similar cell concentration and infected with 10-fold serial dilutions prepared from each archived virus sample. After seven days of incubation, the presence of infected cells was detected by HA, titers were calculated, and they were expressed as log_10_ HAD_50_/mL. In all cases, the difference in virus titer in MA-104 cells when compared to primary swine macrophages conducted with four replicates was approximately 1 log_10_ (with the average among all viruses tested being 1.275 log_10_) (Figure 3).

To further determine the sensitivity of MA-104 cells, the diluted virus used for titrations was tested using a validated real-time PCR (rt-PCR) protocol, as previously described [10] and summarized in Materials and Methods (Figure 3), with an established sensitivity of 1–1.5 genomes (5–50 HAD_50_) per qPCR reaction [13]. Detection of infectious ASFV in MA-104 was more sensitive than that of qPCR by an average of 0.7 logs. This decreased qPCR sensitivity relative to a cellular substrate was previously reported when a comparison was performed using swine macrophages and PCR [13].

## 4. Discussion

Rt-PCR or quantitative PCR (qPCR) is a well-established method for the detection and quantification of large amounts of ASFV clinical field samples. There are also some antibody detection assays that have a colorimetric readout, but the production of antibodies occurs days after ASFV can be found in clinical samples [14]. In ASFV diagnostics, a positive ASFV rt-PCR would still require confirmation of virus isolation before it would be considered a positive sample [15]. Before this report, the only available cell type to validate a positive rt-PCR test was primary swine macrophages. Here, we identified that MA-104 cells could be used to test for the presence of infectious ASFV, with a sensitivity of about one log less (average 1.275 ± 0.34 logs) than primary swine macrophages, but with a greater sensitivity of about one log (average 0.7 ± 0.88) than that of rt-PCR. MA-104 is a commercially available cell line isolated from Cercopithecus aethiops, kidney epithelial cells, commonly used for porcine reproductive and respiratory syndrome virus (PRRS) diagnosis and for simian rotavirus production. MA-104 cells have a doubling time of 72 h [16], do not have any special media requirements, and are easily frozen [16], qualities that are necessary during outbreak situations when a large number of clinical samples may need to be processed to determine if they contain infectious ASFV virus or just ASFV DNA. In addition, the ability of MA-104 cells to form clear HA will aid in the rapid diagnosis of ASFV. In main diagnostic laboratories, fresh swine blood is not readily available, leading to the inability to produce primary swine macrophages, and also the inability to have of a source of red blood cells for observation of HA caused by ASFV. In the absence of a source of red blood cells, MA-104 cells can clearly be stained for the presence of ASFV using anti-ASFV antibodies, which is also useful to confirm the presence of ASFV in some natural isolates that may not form HA due to a mutation in CD2 [11,12,13]. This clear staining of ASFV-infected cells is an advantage over using swine macrophages as a substrate, as swine macrophages have peroxidase activity, which results in a high background in uninfected cells, increasing the possibility of missing a positive result or confirming a false positive one, which is not the case in MA-104 cells that display a very clean background in uninfected cells. Preliminary studies showed that we were able to isolate ASFV from infected blood samples, indicating that MA-104 is an also a good substrate for direct isolation from field samples.
